# Perspectives of Fijian Policymakers on the Obesity Prevention Policy Landscape

**DOI:** 10.1155/2015/926159

**Published:** 2015-08-25

**Authors:** Anna-Marie Hendriks, Mere Y. Delai, Anne-Marie Thow, Jessica S. Gubbels, Nanne K. De Vries, Stef P. J. Kremers, Maria W. J. Jansen

**Affiliations:** ^1^Academic Collaborative Centre for Public Health Limburg, Regional Public Health Service, P.O. Box 2022, 6160 HA Geleen, Netherlands; ^2^Department of Health Promotion, CAPHRI School for Public Health and Primary Care, Maastricht University, P.O. Box 616, 6200 MD Maastricht, Netherlands; ^3^Health Research Unit, Fiji Ministry of Health and Medical Services, P.O. Box 2223, Suva, Fiji; ^4^Menzies Centre for Health Policy, University of Sydney, P.O. Box 170, Sydney, NSW, Australia; ^5^Department of Health Promotion, NUTRIM School for Nutrition, Toxicology and Metabolism, Maastricht University, P.O. Box 616, 6200 MD Maastricht, Netherlands; ^6^Department of Health Services Research, CAPHRI School for Public Health and Primary Care, Maastricht University, P.O. Box 616, 6200 MD Maastricht, Netherlands

## Abstract

In Fiji and other Pacific Island countries, obesity has rapidly increased in the past decade. Therefore, several obesity prevention policies have been developed. Studies show that their development has been hampered by factors within Fiji's policy landscape such as pressure from industry. Since policymakers in the Fijian national government are primarily responsible for the development of obesity policies, it is important to understand their perspectives; we therefore interviewed 15 policymakers from nine Fijian ministries. By applying the “attractor landscape” metaphor from dynamic systems theory, we captured perceived barriers and facilitators in the policy landscape. A poor economic situation, low food self-sufficiency, power inequalities, inappropriate framing of obesity, limited policy evidence, and limited resource sharing hamper obesity policy developments in Fiji. Facilitators include policy entrepreneurs and policy brokers who were active when a window of opportunity opened and who strengthened intersectoral collaboration. Fiji's policy landscape can become more conducive to obesity policies if power inequalities are reduced. In Fiji and other Pacific Island countries, this may be achievable through increased food self-sufficiency, strengthened intersectoral collaboration, and the establishment of an explicit functional focal unit within government to monitor and forecast the health impact of policy changes in non-health sectors.

## 1. Introduction

Fiji is one of the 22 Pacific Island countries (PICs), which have a combined total population of 10,566,500 people [1]. In total, 56.8% of Fiji's population is indigenous Fijian, 37.5% are Indo-Fijian and 5.7% are from other ethnic groups [2]. Fiji contains 332 islands of which one-third are inhabited, covering a total land area of 18,333 square kilometers within 1.3 million square kilometers of the South Pacific [3]. The PICs share regional commonalities: a narrowly based economy, limited national infrastructure, and aid dependence [4]. Nowadays, they also share an epidemic of noncommunicable diseases (NCDs) such as coronary heart diseases and diabetes [5, 6].

In Fiji, 82% of all deaths are attributed to NCDs, contributing to rising health care costs and challenges to economic growth as adults are affected during their most productive years. So, even though infectious diseases have declined and health care has improved, these NCDs have caused life expectancy to stagnate at a low 69 years [3]. One cause of this NCD epidemic is the rapid increase in obesity, which is largely due to poor diets and low levels of physical activity (PA). This not only disproportionally affects Fiji, but also many other small island nations such as Nauru and Tonga where overweight rates vary between 94% and 97% [6]. Although data on regional trends are limited, it is estimated that 56.2% of adults and 14.5% of children in Fiji are overweight [7]. Interestingly, women are significantly more overweight than men, indigenous Fijians are more overweight than Indo-Fijians and urban children are more overweight than rural children. The severity of the obesity epidemic is even more urgent than in many high-income countries, because many obesity-related NCDs go untreated in Fiji. For example, one NCD survey found that 16% of all diabetics between the ages of 25 and 64 years did not know they had diabetes, and among those who knew, 2.1% were not on medication and 32.2% were on medication but had uncontrolled fasting blood glucose. Therefore, diabetes is the most common cause of nontraumatic amputation and the second most common cause of adult blindness in Fiji [8].

Globalization, urbanization, and acculturation lead to an environment that promotes unhealthy dietary intake and sedentary PA patterns. Although daily food intake traditionally consisted of large quantities of relatively healthy starchy roots, green leaves, fish, coconuts, and fruits, dietary studies show that urban populations now consume a high proportion of less-healthy foods (many of which are imported), such as flour, sugar, sugar-sweetened beverages, unhealthy oils, canned fish and meat, and fewer locally produced foods. PA patterns have also become more sedentary, especially in urban centers [9].

The Fijian Ministry of Health and Medical Services has recognized that changing this “obesogenic” environment is important and they aim to change the environment through “*Policies and action on common NCD risk factors through multisectoral collaboration*” [8]. The current national public health policy states that prevention should be comprehensive and multisectoral (i.e., integrated [10]) and explicitly describes policies to address Fijians' diets and PA practices [8]. However, although policy changes are occurring, their development often fails. Barriers related to collaboration between health and nonhealth sectors within government and the society are often seen as the underlying problem [10–14]. For instance, industries were required to collaborate with the Ministry of Health and Medical Services and the Ministry of Finance, Public Enterprises, Public Service & Communications in the implementation of a sugar-sweetened beverage tax policy [15], but they saw it as an unpleasant task because their revenues were based on consumption so discouraging it counteracts their interests.

**Figure 1:**
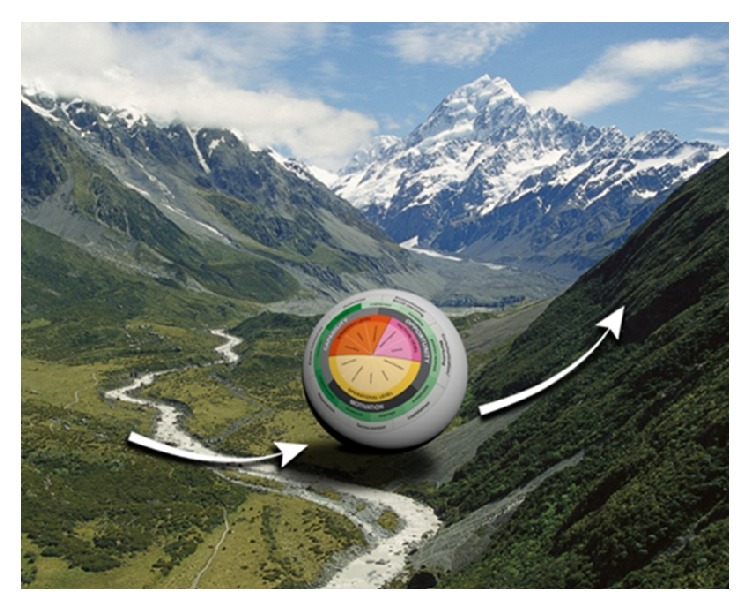
The policy landscape (mountains) and forces (arrows) affecting Fijian policymakers (the ball).

Many countries and especially other small island nations experience similar barriers that are often found in the “policy landscape” (e.g., [12, 13]). The policy landscape includes interacting factors relevant to the policies under consideration, which determine the policymakers'* opportunities* for developing policy. These similarities have led to the growing importance of understanding the policy landscape worldwide. One way to achieve such an understanding is through the application of dynamics systems theory with its commonly used metaphor of the “attractor landscape” [16]. An “attractor landscape” can be seen as the context in which policymakers work (see Figure 1). The ball in Figure 1 can be seen as the Fijian policymakers, where the top of the hill may reflect a policy that has been successfully developed to prevent obesity. For example, in the last years, the Fijian government has implemented several fiscal policy measures targeting the prices of fruits and vegetables, sugar-sweetened beverages, and palm oil [17]. Further implementation and sustenance of the policy will be relatively easy as the ball rolls down the hill. As the ball reaches the “basin,” the behavior of the policymakers is likely to remain stable, as dynamic systems theory assumes that elements in a system prefer a stable state that may mimic habitual routine behavior [16]. To develop new obesity prevention policies, the ball (policymaker) would need to get out of the basin (the status quo) and exert effort (arrows pointing upward) to climb the mountain to arrive at a new obesity prevention policy. However, there will also be forces that make the slope of the hill steeper. An example changes to agricultural and fishery policies that encourage trade, which result in dependence on low-quality food imports, which in turn lead to decreased access to local healthy food. These forces have to compete with economic interests, thus affecting the effort Fijian policymakers must exert to create new policies.

Since policymakers are primarily responsible for developing policy, it is important to understand their perspectives [18]. However, empirical data about the viewpoints of Fijian ministry policymakers about the broader policy landscape is not often described. Instead, most studies in Fiji and other small island countries focus on the barriers present during the development of a single obesity prevention policy measure [12–15]. It is difficult to generalize the results from such studies to develop a set of comprehensive multisectoral policy measures that involve several policy sectors [10]. Currently, we only have a limited view of the policy landscape. As the development of obesity prevention policies is salient for many countries and barriers are not expected to be specific to Fiji, our goal is to describe the shape of the wider obesity prevention policy landscape, using Fiji as an example. This can help in forecasting difficulties that need to be overcome in future attempts to develop obesity prevention policies and can stimulate learning from abroad. Moreover, it is relevant to consider the policy context as an “attractor landscape” in general because it can provide broader insight into the development of obesity prevention policy.

## 2. Methods

### 2.1. Data Collection

We collected data through interviews with policymakers within the Fijian national government. Ethics approval was obtained from the Fiji National Health Research Committee, Ministry of Health and Medical Services, Suva, Fiji. To prepare for the interviews, the policy literature was reviewed and the first two authors (Anna-Marie Hendriks and Mere Y. Delai) brainstormed about obesity prevention policies each ministry could potentially develop. Thereafter, they jointly conducted all the interviews. Anna-Marie Hendriks was affiliated with the Ministry of Health and Medical Services' health policy and research department for five months and Mere Y. Delai was a public health official working at the health policy and research department of the Fijian government in which this study took place.

We used an adapted semistructured interview guide from a previous study on the development of integrated public health policies [19]. Our approach was to first focus on the development of integrated (i.e., comprehensive and multisectoral) public health policies in general and then to focus on integrated policies for the prevention of obesity. We assumed that this approach would reveal more information than narrowing down our focus too early. To arrive at a more accurate interpretation of the data, the two interviewers reflected on each of the interviews afterwards and compared notes. Their reflections were entered into the reports that were also used in the data analysis.

### 2.2. Sample

The 11 ministries that were most likely to affect the development of obesity prevention policies were invited for a one-hour interview. Each ministry received additional information about the study and was asked to select a policy representative. Our goal was to interview at least one representative from each ministry; only when interviewees indicated that other representatives could complement their interview did we opt to speak with extra interviewees from the same ministry.

All the ministries were willing to participate, but two declined because of time limitations or because the ministry said that no representatives were available during the research period (January–May 2014). In total, 15 representatives from nine different ministries participated (Table 1). Representatives included three Permanent Secretaries, three Deputy Secretaries, three Departmental Managers, and six operational-level policymakers. Operational-level policymakers were often interviewed because they could complement the information given by their managers or secretaries. We attained data saturation regarding the factors in the policy landscape after these 15 interviews.

### 2.3. Data Analysis

All interviews were summarized and coded using MaxQDA software [20]. Because we were interested in the policy landscape, we coded themes that provided barriers or facilitators within the “opportunities” of the Fijian policymakers. These opportunities were defined as “*factors that are lying outside the individual Fijian policymaker and make the development or implementation of obesity prevention policy possible or prompt it*” [21]. The attractor landscape metaphor [16] was used to aid thinking about the difference between distal and proximal opportunity factors and to provide codes. When opportunities were more distal to policymakers, we coded them as forces that steepen or flatten the slopes of the mountains in Fiji's policy landscape. When factors were more proximal to the policymakers, we coded them as factors within the ball (i.e., as efforts the Fijian policymaker should invest in to reach the top of the mountain; Figure 1). Data was categorized under a code after consensus between the two researchers was reached. Even though we also recognized the importance of motivational and capability related factors during policymaking [21, 22], they will not be discussed in this article.

## 3. Results

We will now give an overview of barriers to and facilitators of opportunities for Fijian policymakers. Results do not include data from the literature, but only report the perceptions of the interviewed Fijian policymakers. Sections 3.1 through 3.5 describe policy landscape factors, while Sections 3.6 through 3.8 describe factors within the ball.

### 3.1. Poor Economic Situation

Four interviewees mentioned that because Fiji has high poverty rates, it is important to be extra careful to avoid unintended policy effects on economic development and income. For example, one interviewee mentioned that they cannot implement a ban on food vendors selling around schools because this could push families into poverty:

*It is very difficult, if you prohibit marketing of food around schools. For example, there is one family who sells in front of a school. Each day, children buy sweets there. If we prohibit it, this family will lose their income. And these are the poorest families.*  (Interviewee from the Ministry of Health and Medical Services)

### 3.2. Low Food Self-Sufficiency

A barrier often mentioned by interviewees was Fiji's low food self-sufficiency due to a poorly organized agriculture sector. Although lots of local food processing is done (e.g., by Flour Mills of Fiji and Punjas), interviewees often mentioned that there is insufficient production of healthier and/or fresh food to meet the dietary needs of all Fijians. Therefore, interviewees often mentioned that Fijians were increasingly reliant on highly processed and often imported foods. Although interviewees also mentioned the role of local food processors in the onset of obesity, imported foods were considered to be one of the main causes of the current obesity epidemic. Imported foods were perceived often to be highly processed and to have high fat and sugar percentages. One interviewee said that reliance on such less-healthy foods was greater in urban areas due to the absence of land for subsistence agriculture (i.e., self-sufficiency farming). This interviewee explained that because, for decades, most Fijians lived by growing food only for their own needs, there was no need for rural Fijians to develop a more commercially oriented agriculture sector. As a result, these farmers have a poor attitude towards production for sales (i.e., commercial farming):

*Extension officers (those who train the farmers on how to commercially farm) visit localities, but encounter a difficult mentality. When farmers want to drink kava and are not interested in farming more than they are used to, why would they? Export-oriented production and even internal market promotion is limited.* (Interviewee from the Ministry of Agriculture, Rural and Maritime Development and National Disaster Management)

He added that it is difficult for farmers to develop competitive food prices and a well-organized profitable agriculture sector; the poor infrastructure in many farming areas leads to high transportation costs, making it difficult to transport products from villages to farms, from farms to markets, and from the outer islands to the main islands. Moreover, two interviewees explained that it is challenging to develop competitive food prices because Fiji has a small market (881,065 citizens in 2013), which makes it difficult to compete with multinationals on worldwide access to markets.

### 3.3. Framing of Obesity

Although most interviewees cited the changing food supply (i.e., more processed foods containing higher fat and sugar percentages) as the main cause of obesity, they also frequently related the issue to changes in Fijian culture and Fijians' individual eating and PA preferences. Six interviewees explained that in the Fijian culture, “big is beautiful,” suggesting that obesity is often seen as desirable. Almost all interviewees mentioned that even though there seems to be an ongoing mixed preference for both robustness and thinness with Fijian society, the food industry plays an important role in reinforcing the idea that Fijians are not interested in losing weight or eating healthily. Three interviewees seemed to have adopted this framework from the food industry; they took the lack of impact from price increases on sugar-sweetened beverages as evidence for the legitimacy of this framework. Interviewees explained that Fijians would only look at the* present*. This different time perspective (i.e., “vakaviti”) reduces interest in preventing* future* consequences (e.g., weight gain) and therefore the impact of, for example, a sugar tax:

*There did not seem to be a decrease in consumption of the products (referring to sugar-sweetened beverages and tobacco) that had recently increased in price due to tax policies. This is surprising given the poverty rate. Not the price determines consumption, but awareness. Thus, in the end, the poor get poorer due to increasing food prices and therefore there needs to be a balance.* (Interviewee from the Ministry of Finance, Public Enterprises, Public Service & Communications)

According to these interviewees, this problem is also apparent in the lack of effects from several tobacco prevention policies: tobacco price increases did not lead to a decrease in tobacco consumption in Fiji. In addition to this cultural framework, all the interviewees framed obesity as an individual health problem caused by poor food and PA choices. For example, one interviewee said that many Fijians perceive the preparation of breakfast to be too time-consuming an activity because they traditionally used to cook breakfast. Many interviewees also reported a poor attitude towards PA in daily living. Therefore, all the interviewees said that Fijians should be made aware of these practices; only then would obesity prevention policy have an impact. Most interviewees recommended interventions based on individual determinants of obesity, such as increasing understanding that breakfast can be quick and does not necessarily require cooking for hours or that PA is not bound to sport activities but can be integrated into daily living by, for instance, walking to the office rather than taking a cab.

### 3.4. Power Inequalities

A constraining factor often mentioned by interviewees was the power inequality between Fiji's government and international actors such as the World Trade Organization (WTO) and the food industry. For example, one interviewee from the trade sector mentioned that the WTO has a clear liberalization agenda that has been formalized in trade agreements that prohibit member states from imposing barriers to free trade. Two interviewees mentioned that it would therefore be difficult to develop policies that limit the import of unhealthy food. They explained that unless there is clear evidence that imports can damage the country in terms of, for instance, safety or health, the Fijian policymakers were concerned about the possibility that they could be taken to some form of international dispute settlement or arbitration for banning unhealthy foods. A major concern was the potential cost of such action for the Fijian government. Interviewees perceived the lack of resources as a factor that makes Fijian policymakers powerless.

Moreover, there was a perception that there is still scarce evidence that the use of* specific* products (e.g., Coca-Cola instead of sugar-sweetened beverages in general) leads to obesity. So even if the resources existed, it might still be hard to provide evidence to defend policies in an international context. Furthermore, interviewees mentioned that multinationals sometimes use their monopoly as providers of certain products to Fiji to hamper the development of obesity prevention policies. The food industry could, for example, threaten to leave Fiji's market if the Fijian government imposed more stringent food import policies. Another interviewee mentioned that the food industry could hamper implementation of television marketing policies:

*Developing policies that limit the exposure of children to advertising of unhealthy food products is difficult because the big food producers sponsor most programs and without such sponsorship it is difficult to produce television.* (Interviewee from the Ministry of Health and Medical Services)

One interviewee added that the food industry sometimes uses the lack of clarity around the legal definition of a child to postpone child marketing regulations. In response, three interviewees use United Nations conventions as “back-up” legislation to form a basis for asserting the right to good quality and healthy food in obesity prevention policies. By using such human rights documents, some of the power inequality could be restored:

*United Nations conventions emphasize the right to food, access to food. Food security is part of national security because it protects citizens from lack of food or a low quality of food. In this regard, the Ministry of Agriculture plays a big role forming the basis for the right to good quality and healthy food.* (Interviewee from the Ministry of Immigration, National Security and Defence)

### 3.5. Lack of Evidence

There is a lack of evidence about what works for Fiji's relatively young population. One interviewee said that the development of NCDs must start during childhood because the youngest generations suffer from NCDs and the age of deaths in this cohort is very early (16% live beyond 50 years of age and only 8% live beyond 60 years). However, this interviewee said that most policy evidence is derived from countries with relatively older populations and is thus not suitable for Fiji, a place in which 60% of the total population is under 30 years of age. For instance, to increase the font size on food labels, one would need to obtain sufficient evidence that food labels actually affect the consumer behavior of young Fijians. Many interviewees said that this gap in evidence is likely to persist due to a lack of resources within the Fijian government to facilitate policy evaluation. Therefore, it is currently difficult to determine the feasibility and effect of certain obesity prevention policies and to convince “antiobesity” policy actors that they have responsibility in obesity prevention:

*The most important question for the unit is to ask, ‘Will it make a difference?' This argument drives changes in the food industry. If you can make it very clear that it will make a difference, chances are bigger that regulations will be implemented. Otherwise you can expect a lot of resistance from those who implement the policies.* (Interviewee from the Ministry of Health and Medical Services)

This makes it difficult for Fijian policymakers to convince or force the food industry to take a role in obesity prevention. On a more positive note, many interviewees mentioned the significant role policy brokers from universities play in generating evidence.

### 3.6. Limited Resource Sharing

Two interviewees from the Ministry of Health and Medical Services explained the Fijian government has limited policy resources and that sharing resources and integrated obesity prevention policy are essential. All interviewees explained that resource sharing is difficult because nonhealth policy sectors within the Fijian government, nongovernmental organizations (NGOs) in the health, food, and beverage industry, the WTO and policy implementers often have goals other than obesity prevention and that going against these goals would be difficult. For example, two interviewees said that the promotion of exports is high on the government's agenda due to the import-export imbalance. Due to very strict EU regulations, one of these interviewees mentioned that the Fijian government needs to focus most resources on controlling exports:

*Export policies are also determined and controlled by the Ministry of Industry and Trade. They determine the standards with which Fiji's products should comply in case they want to export with other countries. Since the EU has very high and strict standards, Fiji should be very careful; otherwise they might lose a market.* (Interviewee from the Ministry of Health and Medical Services)

This interviewee said that, as a result, there are fewer resources available to control the import of health-damaging products. The related political risks and costs are often so high that it is most often avoided, even though, in theory, it would be possible to amend other policy sectors' agendas. Due to the notable difficulties in aligning policy agendas, most interviewees mentioned that extra effort is required to develop a shared agenda. Two interviewees explained that it is difficult even to achieve this, as Fiji has a small workforce charged with developing policy. They mentioned that building partnerships is an alternative strategy to overcoming resource scarcity. However, they believe that the quality of relationships with health NGOs is poor:

*There are no collaborating NGOs on health nutrition; the only one that is present is funded by Vodafone. They, however, do not align their work with that of the National Food and Nutrition Centre; for example, they approach the same schools that are also health-promoting schools, instead of approaching different schools.* (Interviewee from the Ministry of Health and Medical Services)

One interviewee mentioned that the Ministry of Strategic Planning, National Development and Statistics did not invest in building partnerships with private organizations or NGOs at the beginning of the policy cycle. This interviewee perceived that this hampered policy implementation due to poor public-private partnerships. It was expected that investing in building partnerships at the beginning of the policy cycle could remove barriers for resource sharing with NGOs or other private organizations. Currently, interviewees perceive the feasibility of most obesity prevention policies to be low.

However, one interviewee mentioned that the recent shift from a top-down towards a bottom-up policymaking style contributes to building partnerships. According to this interviewee, the current government involves stakeholders in conversations more frequently and empowers citizens since it realizes it cannot achieve its goals alone. Collaboration with citizens and also with other governmental departments is considered to be key in the current government. It is expected that enforcement mechanisms would then require fewer resources because policy implementers would be more likely to accept the policy change. In other words, enforcement mechanisms and resources would only be required if policy change were not accepted.

Some interviewees said that the biggest challenge in developing certain policies is related to the difficulties in aligning policy implementers' belief systems. For example, if teachers would not align their academic goals with health goals, extra health inspection staff would then need to be hired to check whether teachers were complying with health promotion requirements such as implementing PA classes. Obtaining such extra resources would be difficult due to the limited budget for preventive public health policies. Some interviewees added that Fiji's communal culture makes it difficult to work without official stringent enforcement mechanisms because Fijians would not easily report noncompliance.

Another positive contribution to resource sharing is the work of policy entrepreneurs and policy brokers within and outside the Fijian government. They promote a strong integrated vision around obesity prevention, help the current government recognize the problem, are active in overcoming incompatible policy priorities in other sectors, and are active when a window of opportunity opens. As the main ministerial office, the Prime Minister's Office is tasked with monitoring the government's implementation activities, along with the Ministry of Health and Medical Services' Wellness Centre, Food Unit and National Food and Nutrition Centre. These were often mentioned as the most important policy* entrepreneurs* within government. Outside government, the Pacific Research Center for the Prevention of Obesity and Non-Communicable Diseases (C-POND) and the Secretariat of the Pacific Community (whose Public Health Division is dedicated to improving the health of Pacific Islanders) were often mentioned as entrepreneurs. Policy* brokers* were often people affiliated with universities who are involved in creating conditions to establish network contacts (e.g., through workshops) and policy evaluation.

### 3.7. Window of Opportunity

One interviewee referred to a recent UN meeting as a “window of opportunity” to facilitate and progress development of obesity prevention policy. In 2011, the Fijian government attended a United Nations High-level Meeting of the General Assembly on the Prevention and Control of Non-communicable Diseases in New York, which increased its willingness to invest in obesity prevention policy. Other interviewees referred to the increased recognition of obesity by the current government as a direct outcome of the Pacific Island countries' Health Ministers meeting. Another window of opportunity factor noted by policymakers from the National Food and Nutrition Centre was the flexibility of the current regime (before 2014 elections), which gave them the opportunity to present during cabinet meetings:

*We should educate the Cabinet about the nature of public health; health is not a sole responsibility of the Ministry of Health but is affected by the policies of other sectors. Currently, most cabinet members have a vague understanding of what health contains. Education by staff of the NFNC (National Food and Nutrition Centre) is more effective than the Minister of Health himself, since staff is much more engaged on the topic.* (Interviewee from the Ministry of Health and Medical Services)

Most interviewees also viewed the 2014 election (which was under preparation during the time these interviews took place) as a window of opportunity for presenting important issues pertaining to the welfare of the wider population, such as the obesity prevention policies. The general idea stemmed from the fact that during the preelection period during which the interim government's decision making was centralized, it could be easier to pass progressive policy proposals (e.g., making child marketing rules stricter).

### 3.8. Intersectoral Governance Structures

The most proximal factor (i.e., that which Fijian policymakers can most easily affect) is the development of mutual agreements and policies between Fijian ministries to strengthen intersectoral collaboration for obesity prevention policies. One interviewee referred to a memorandum of understanding:

*Recently, more collaboration has been initiated with the Ministry of Health. We were both on our way to a symposium in Brisbane and met at Nadi airport. In Brisbane, we came to the idea that we wanted to apply for a grant that the university gave, for those who wanted to improve health and sports. We did not get the grant, but after that the relationship (with the Ministry of Health, Wellness Centre) was established (referring to a memorandum of understanding in which the intention to collaborate was formalized). This collaboration started in 2012.*  (Interviewee from the Ministry of Youth and Sports)

The Ministry of Health and Medical Services' Public Health Act is currently being reviewed after almost 80 years. In recognition of the role the environment plays in the onset of NCDs, a recommendation about the development of intersectoral policy measures to prevent NCDs was part of the submission towards the reviewed act. Furthermore, some interviewees mentioned that the military regime (which was recently reelected) established a national roadmap for change based on the People's Charter and a National Strategic Plan. Interviewees said that these documents are facilitative because the National Strategic Plan is implemented through a system of key performance indicators that recommend intersectoral collaboration during policy developments. Although interviewees said that this new intersectoral collaboration reporting framework was difficult to implement, this is facilitative for intersectoral collaboration in theory. Interviewees also added that intersectoral governance structures could be improved by developing an explicit Health in All Policies strategy. Within this strategy, it was recommended that a formal position for an official to implement the strategy be created. A person in such a position would need to be active in building networks for obesity prevention and also monitor and forecast the health impact of policy changes in nonhealth sectors.

## 4. Discussion

The aim of our study was to describe the perspectives of Fijian policymakers on the obesity prevention policy landscape. We illuminated Fijian policymakers' efforts to develop obesity prevention policy (i.e., reach the top of the mountains) and described how several factors make it more difficult. We will now discuss four themes that may make the obesity prevention policy landscape more conducive towards the development and sustained implementation of obesity prevention policies in Fiji and other PICs.

Firstly, Fijian policymakers need to* integrate* health priorities with economic priorities and* share resources*. For example, Thow et al. [15, 23] outlined how increasing taxes on sugar-sweetened beverages contributes to public health and to government revenues. However, full integration of economic interests with public health might be challenging since Fiji is a country with a transitional economy and economic growth is based on consumption [24–27]. To overcome this potential integration barrier, a health impact assessment may be used. Such an assessment could clarify that the long-term costs of obesity could overshadow economic wins [25]. Furthermore, the sharing of resources between policy sectors within the Fijian government (i.e., a factor within the ball) may be increased if health policymakers strategically plan for agenda setting, identify priorities and synergies in nonhealth sectors, and base proposals on existing legislative mechanisms where possible [15].

Moreover, intersectoral advocacy coalitions might be developed through early engagement with stakeholders outside the health sector [15]. Policymakers from the Ministry of Health and Medical Services can be trained to detect a window of opportunity and increase advocacy during cabinet and international meetings. Policy entrepreneurs and brokers such as the Ministry of Health and Medical Services' Wellness Centre, the World Health Organization, and C-POND can assist in generating policy alternatives. In combination with focusing events such as the United Nations High-Level Meeting of the General Assembly on Non-Communicable Diseases, a policy window might open [28–30]. Furthermore, managers might assist policymakers in reframing health goals in the terminology of nonhealth policy sectors and stimulating awareness of public health in nonhealth policy sectors [19]. Additionally, efforts to integrate health with nonhealth sectors might become more sustainable if intersectoral governance structures are institutionalized by the Fijian government. A feasible first step to achieve this might be to establish a national Health in All Policies strategy, accompanied by a formal position to monitor and forecast the health impact of policy changes in nonhealth sectors.

Secondly, if Fiji's food self-sufficiency and food security can be increased, Fijians might become less dependent on international multinationals or neighboring countries that supply food products that contribute to obesity. For example, in the context of liberalized trade, New Zealand exports high-fat mutton flaps and tobacco to PICs [4]. Although New Zealand also provides support for NCD prevention, these products make it very difficult for Fiji to prevent obesity. Policies that focus on local food production, improved agricultural production through promoting new technologies, crop diversification, capacity building activities, dissemination of information, and monitoring could therefore facilitate the development of obesity prevention policies [17, 31–33].

Countries with better economies can help Fiji in this regard by voluntarily limiting their export of health-damaging products (i.e., stop dumping) and assisting Fiji in strengthening local enterprises and farms, human resources, and technological development. Other countries should recognize that the comparative advantage of Fiji (and other PICs) on international markets is low; thus its remoteness, geography, and limited natural resources make it difficult to develop competitive export prices for their products (including food) [34–36]. Thow et al. [14] therefore suggested that the health sector should be actively engaged in the negotiation of trade agreements to support healthier trade in the region. Negotiators should understand the implications of trade for all sectors of the economy and identify opportunities to improve the terms of negotiation for their countries.

Thirdly, Fiji's obesity prevention policy landscape might become more conducive to change by illuminating the “obesity framing contest.” Some interviewees adopted the frame from the food industry but were not aware that such framing decreased the food industries' responsibility in obesity prevention. These interviewees emphasized education as the key solution to obesity, while the actual causes of rapidly increasing obesity rates seem to be primarily related to the changing food supply. Therefore, making the interests of the frame's sponsors transparent might help in reducing the hampering effects of obesity framing [37]. At the same time, however, policymakers should recognize that Fijians (and also most other Pacific Islanders) traditionally perceive a large body size as desirable and an indicator of not only wealth, but also of being cared for and respected [38, 39]. Moreover, culturally determined timeframes might influence the extent to which Fijians look into the future;* preventing* obesity might be less successful if it requires activities that could be instrumental in* future* outcomes while “typical Fijian” timeframes are shorter [40, 41]. This makes it important for policymakers to understand how sociocultural factors influence eating, activity, and body size [42]. The Translational Research for Obesity Prevention in Communities (TROPIC) project is already active in turning knowledge about the sociocultural factors of obesity (found in C-POND) into obesity prevention policies [43]. Supporting the work of such researchers therefore remains important.

Fourthly, the lack of evidence about the efficacy of policies for Fiji's relatively young population hampers the development of obesity prevention policy. Evidence could legitimize policies, especially when they fit with national norms, values, practicability, feasibility, and affordability without excluding certain groups (i.e., evidence-informed policy) [44]. Although the TROPIC project [43] has greatly increased the evidence and legitimation, this is still scarce [45].

*Strengths and Limitations of the Study*. A strength of this study is that we were able to conduct face-to-face interviews with representatives from a wide range of ministries, resulting in heterogeneous and in-depth data. Therefore, our data offers a broad view of the policy landscape. A limitation is that the data was cross-sectional and the interviews were not triangulated with focus groups or questionnaires. Moreover, we only interviewed one to four interviewees per ministry and we could not interview any representatives from some relevant ministries during the research period. Further, even though we assured interviewees that data would be anonymized, they might have felt pressure to give socially desirable answers (i.e., they knew the interviewers were working at the Ministry of Health and Medical Services). Finally, it remains challenging to generalize results from Fiji to countries that are not PICs because of their specific characteristics.

## 5. Conclusions

Fijian policymakers clearly invest in obesity prevention policies, but their efforts are often hampered by the policy landscape. Policy entrepreneurs and brokers, researchers, and international actors such as the food industry, the WTO, and countries with better economies in general can support the Fijian government in reducing power inequalities and increasing food self-sufficiency. Establishing a national Health in All Policies strategy and intersectoral governance structures may be a suitable first step towards achieving this goal.

## Figures and Tables

**Table 1:** Interview sample, F = female, M = male.

Ministry	Role in obesity prevention	Participants (*n*) – total (15)
Ministry of Youth and Sports	Sport and youth policy	Official 1 (M)

Ministry of Agriculture, Rural and Maritime Development and National Disaster Management	Food self-sufficiency, assistance schemes related to poverty alleviation, and farming	Official 2 (F) and official 3 (M)

Ministry of Immigration, National Security and Defence	Food security, safe PA environment	Official 4 (M)

Ministry of Industry & Trade and Tourism	Limiting import of unhealthy products	Official 5 (F)

Ministry of Health and Medical Services: NFNC, Food Unit, Wellness Centre, Policy Unit	Health education and promotion, NCD strategy	Officials 6 (F), 7 (M), 8 (M), and 9 (M)

Ministry of Education, Heritage & Arts & National Archives of Fiji	Health-promoting schools	Official 10 (M)

Ministry of Finance, Public Enterprises, Public Service & Communications: Revenue Section	Taxes on sugar-sweetened beverages, unhealthy foods	Officials 11 (F), 12 (M), and 13 (M)

Ministry of Local Government, Housing and Environment: Suva City Council	Designing an attractive environment for PA	Officials 14 (M) and 15 (M)
